# How Intractability Spans the Cognitive and Evolutionary Levels of Explanation

**DOI:** 10.1111/tops.12506

**Published:** 2020-06-04

**Authors:** Patricia Rich, Mark Blokpoel, Ronald de Haan, Iris van Rooij

**Affiliations:** ^1^ Philosophy Department University of Hamburg; ^2^ Philosophy Department University of Bayreuth; ^3^ Donders Institute for Brain, Cognition, and Behaviour Radboud University; ^4^ Institute for Logic, Language and Computation University of Amsterdam

**Keywords:** Levels of explanation, Rationality, Heuristics, Modularity, Evolution, Intractability

## Abstract

The challenge of explaining how cognition can be tractably realized is widely recognized. Classical rationality is thought to be intractable due to its assumptions of optimization and/or domain generality, and proposed solutions therefore drop one or both of these assumptions. We consider three such proposals: Resource‐Rationality, the Adaptive Toolbox theory, and Massive Modularity. All three seek to ensure the tractability of cognition by shifting part of the explanation from the cognitive to the evolutionary level: Evolution is responsible for producing the tractable architecture. We consider the three proposals and show that, in each case, the intractability challenge is not thereby resolved, but only relocated from the cognitive level to the evolutionary level. We explain how non‐classical accounts do not currently have the upper hand on the new playing field.

## Abstract 2

This paper focuses on the cognitive/computational and evolutionary levels. It describes three proposals to make cognition computationally tractable, namely: Resource Rationality, the Adaptive Toolbox and Massive Modularity. While each of these proposals appeals to evolutionary considerations to dissolve the intractability of cognition, Rich, Blokpoel, de Haan, and van Rooij argue that, in each case, the intractability challenge is not resolved, but just relocated to the level of evolution.

## 1 Introduction

Cognitive scientists face a key explanatory challenge: How can cognition both fit the demands of our complex world and still be computationally tractable? To understand the weight of this challenge, it is useful to explicate the nature of the problem solved by a cognitive agent who successfully navigates its world. In general terms, we can cast the problem as an ability to select an action that is rational (or otherwise “good”) for the agent, in each situation it can face. Since agents cannot condition their behavior directly on the world, each possible situation gets represented by the agent, and then the agent chooses among its available actions based on this representation. Let us state this ability in terms of its input domain and the presumed output that it produces:

Rational Action

*Input:* A representation *r*(*s*) ∈ *R* of a situation *s* ∈ *S* and candidate actions *A*.

*Output:* An action *a* ∈ *A* such that *a* is rational with respect to *r*(*s*), *S* and *A*.

This computational problem is known to have computationally intractable special cases. This means that the computation consumes astronomical computational resources for all but trivially small inputs; more formally, the problem is NP‐hard or worse (Arora & Barak, 2009; Garey & Johnson, 1979; van Rooij, Blokpoel, Kwisthout, & Wareham, 2019). For instance, classical accounts of rational action in cognitive science (Bossaerts, Yadav, & Murawski, 2019; Fodor, 2001; Savage, 1954) propose that the problem is solved by building (probabilistic) models representing our (incomplete) knowledge of the causal structure of the world and performing computations over these models in order to decide which actions to take in which situations. Such models may represent, for instance, the probabilities with which different candidate actions bring about more or less desirable outcomes. The chosen action is then one that maximizes expected utility. This yields the following special case of the general Rational Action problem:

Classically Rational Action

*Input:* A representation *r*(*s*) ∈ *R* of a situation *s* ∈ *S* and candidate actions *A*.^1^

*Output*: An action *a* ∈ *A* that maximizes (expected) utility relative to *r*(*s*) and *A*.

Classical rationality (CR) poses a computationally intractable problem; that is, there exists no general tractable (polynomial‐time) process that, for any given situation, selects an action with maximum expected utility (Bossaerts et al., 2019). In other words, CR fails to give a tractable account of how cognition yields real‐world action.^2^ Simon (1978) quickly recognized and emphasized the relevance of tractability for CR once the technical notion was introduced (he had argued even earlier that the computational resources required for CR were problematic). Since then, this setback has been used to argue against CR and to motivate alternative approaches to rationality.

One popular strategy pursued by competing research programs is to make an appeal to evolution as a way to dissolve the intractability of the action‐selection problem. This strategy has essentially opened up a second playing field for the competitors. The two fields represent different, but compatible and complementary, (sets of) levels of explanation, as we now explain.

Commonly, cognitive research programs develop their theories by working at one or more of Marr's (1982) levels of analysis. We label this kind of research generally as occurring at the cognitive level, referring collectively to Marr's *computational*, *algorithmic*, and *implementational* levels. In this paper, we characterize approaches to cognition in terms of the problems they take to be solved by the mind: We state the computational‐level theories that they imply. However, a theory will be plausible just insofar as we could also find algorithms to compute solutions to those problems (as emphasized by Gigerenzer, 2019), and insofar as those algorithms could be implemented in our brains (N.B. this yields tractable computability as a minimal constraint; van Rooij, 2008). The computational level can be understood as describing *what* cognition does, while the algorithmic and implementational levels describe *how* it does this. Different research programs tend to focus on different levels in this sense, but a complete explanation of cognition would ideally fill in the details at each level; no single level tells us enough (Bechtel & Shagrir, 2015; R. Cummins, 2000).^3^

Although proponents of every theory of cognition would point to empirical evidence for support, progress at the cognitive level is still far from sufficient to settle the question of which is/are correct. We are not at the point where the major theories discussed in this paper can be confirmed or falsified by truly decisive empirical implications. Approaches largely try to distinguish themselves based on their theoretical merits, such as positing tractable computations. One merit widely claimed and emphasized is the evolvability of the posited tractable cognitive architecture. This means that the debate now incorporates another level, namely the evolutionary level.

Tinbergen's biological levels of explanation (Tinbergen, 1963) will be less familiar to cognitive scientists than are Marr's levels, but the debate is now implicitly situated within them. Bateson and Laland describe these levels as reflecting “four questions about any feature of an organism: what is it for [survival value]? How did it develop during the lifetime of the individual [ontogeny]? How did it evolve over the history of the species [evolution]? And, how does it work [causation]?” (2013, p. 1; names are Tinbergen's). Marr's and Tinbergen's systematizations are compatible, and we can understand Tinbergen's biological levels as encompassing the cognitive level; cognition is, after all, part of the biological world.

From the biological perspective, we can take the cognitive level as a whole to correspond to Tinbergen's causation level: Explaining what *specifically* cognition does—and how—falls under the scope of explaining how cognition works in a more general sense. One might have the intuition that only the algorithmic and implementational levels explain how cognition works, while the computational level tells us something else. While computational‐level characterizations often go along with a story about the survival value of the computation, however, what we refer to as the computational level need not itself provide such an explanation (Egan, 2018; van Rooij, Wright, Kwisthout, & Wareham, 2018). Note that the computational‐level characterizations of the competing theories that we provide throughout the paper illustrate this: These theories hypothesize *how* cognition works in a salient sense by hypothesizing *what* problem it solves with a greater degree of precision.^4^ With respect to this expanded explanatory landscape, the debate regarding cognitive architectures now includes both the cognitive (causal) and evolutionary levels.

Explanations offered at the two levels are complementary (just as with Marr's levels), and in principle evolutionary arguments could prove important in assessing approaches to cognition (see e.g., Cummins, 2000, pp. 135–136; De Jong, 2002, p. 452). Regarding tractability, considering evolution could be important to understanding how cognition can be tractable. It is important, however, that appeals to evolution not give a false impression of explanatory strength. Our concern here is that pushing responsibility for producing a tractable cognitive architecture to evolution may appear to solve the tractability problem, but if evolution itself could not do the work attributed to it, then the appeal to evolution merely relocates and obscures the tractability challenge, without in any way solving it. Because of this, it is essential to verify that when approaches do make use of a levels shift to solve a problem, the complete evolutionary–cognitive package is coherent.

In this paper, we consider three distinct proposals. First, the *Resource‐Rationality* (henceforth, RR) approach proposes that agents optimize action selection relative to resource constraints (Lieder & Griffiths, 2019). Essentially, RR proposes that agents solve a new version of the expected utility maximization problem, maximizing only relative to limited beliefs which are formed on the basis of limited computational resources. Second, the *Adaptive Toolbox* (AT) theory maintains that agents rely on simple heuristics which are well‐tailored to their contexts of application. Lastly, the *Massive Modularity* (MM) thesis is that cognition is *entirely* the product of highly specialized modules (Sperber, 1994; Tooby & Cosmides, 1992).

For RR, AT, and MM alike, the evolutionary explanatory strategy postulates that our cognitive architecture is the product of evolution and tractable‐by‐design. Here, “tractable‐by‐design” refers to the fact that these cognitive architectures have been defined to guarantee tractability, that is, for any situation the agent can encounter, its cognitive computations are guaranteed to be tractable (cf. van Rooij, Blokpoel, de Haan, & Wareham, 2019). The specific design is what separates the three theories. The appeal to evolution may avoid the intractability problem at the cognitive level, but it leaves unexplained how the postulated architecture type could be tractably evolvable. We use computational complexity results to show that, contrary to intuition, intractability remains a problem at the evolutionary level for all three of these approaches. Our results are important because, while we rarely know exactly how evolution proceeded, they establish that offloading explanation to evolutionary processes is insufficient to meet the important explanatory challenge that we started with.

### 1.1 Overview

This paper is organized as follows. Section 2 explains how RR, AT, and MM seek to avoid CR's intractability at the cognitive architecture level and the role played by the evolutionary level in their argumentation. In Section 3, we define a general adaptation problem to be solved by evolution, and we show how each of the three approaches posits that evolution solves a special case of this problem. We prove, however, that in each case, the problem is intractable. In contrast, CR is tractably evolvable. Section 4 concludes.

## 2 The evolutionary strategy

### 2.1 Proposed sources of cognitive‐level intractability

There is wide agreement that CR poses an intractable problem to agents, and at least within cognitive science, agreement that we need to develop explanations that avoid this problem. Specific diagnoses of the source of the problem differ, however, and we can characterize prominent research programs as responses to these different diagnoses. We focus here on RR, AT, and MM, considering them in turn.

RR is inspired by the Rational Analysis approach championed by Anderson (1990; see also Anderson & Matessa, 1990; Chater & Oaksford, 1999). Anderson proposed thinking of cognition as a set of optimal solutions to adaptation problems. From this perspective, in order to explain cognition at the computational level, one should start by hypothesizing a cognitive behavioral function that is optimal given the agent's goals and adaptation environment. Specifically, he proposed the following iterative six‐step procedure (called *rational analysis*):

1. Specify precisely the goals of the cognitive system.
2. Develop a formal model of the environment to which the system is adapted.
3. Make minimal assumptions about computational limitations.
4. Derive the optimal behavioral function given 1–3 above.
5. Examine the empirical evidence to see whether the predictions of the behavioral function are confirmed.
6. Repeat; iteratively refine the theory.

Step 3 was important to Anderson; he went on to integrate the computational‐level Rational Analysis with his work at the implementation level of analysis—hence developing ACT‐R—and took constraints seriously in so doing (Anderson, 1996).^5^ His successors have often ignored Step 3, however, and “minimal assumptions” has sometimes been interpreted as “no assumptions at all” (Chater, Oaksford, Nakisa, & Redington, 2003, p. 69; van Rooij et al., 2018). Rational Analysis therefore retains a rather classical notion of rationality and, as a result, may yield intractable behavioral functions. The guiding premise of RR is that resource limitations must be built in from the start, in order to avoid this. The Rational Analysis procedure is valuable because it provides a source of specific hypotheses, but it goes too far in proposing that agents behave optimally without qualification. We cannot expect outright‐optimal behavior because finding optima requires far more resources than are available. By directly accounting for costs, however, RR aims to preserve the benefits of an optimization approach while positing only affordable—hence feasible—computations.

In order to state RR's theory precisely, the cognitive problem it poses can be broken down as follows (based on Griffiths, Lieder, & Goodman, 2015):

Resource‐Rational Action (1)

*Input:* A resource‐rational computed representation *r*′(*s*) ∈ *R* of a situation *s* ∈ *S* and candidate actions *A*.

*Output*: An action *a* ∈ *A* that maximizes (expected) utility relative to *r*′(*s*) = Resource‐Rational‐Belief( C, s, A).

Resource‐Rational Belief

*Input:* A set of all possible sequences of computations *C*, a situation *s* ∈ *S*, and candidate actions *A*.

*Output*: A representation^6^ *r*′(*s*) produced by a sequence *c* ∈ *C* that as well as possible trades off (expected) utility of the maximum‐(expected)‐utility action *a* relative to *r*′(*s*) against the cost of the computation *c*.

So‐called optimization under constraints theories such as RR have been criticized by competing research programs for various reasons. An important objection is that building costs into the optimization problem does not make optimization feasible; instead, it makes it harder, so the problem remains intractable. As Gigerenzer, Hertwig, and Pachur (2011, p. xx) write, optimization “becomes more demanding mathematically with each constraint added.”

To address this problem, RR theorists have proposed that the potentially intractable resource‐rational decision problem is approximately (but again resource‐rationally) solved via heuristics. This requires them to postulate an additional process that yields such heuristics (Lieder & Griffiths, 2019). The idea is expressed by the following pair of computational‐level characterizations:

Resource‐Rational Action (2)

*Input:* A resource‐rational computed representation *r*′(*s*) ∈ *R* of a situation *s* ∈ *S*, candidate actions *A*, and a decision process *h* which is the output of Resource‐Rational‐Heuristic.

*Output*: An action *a* ∈ *A* selected by *h*.

Resource‐Rational Heuristic

*Input:* A set of all possible heuristics *H*, a situation *s* ∈ *S*, and candidate actions *A*.

*Output*: A heuristic *h* ∈ *H* that yields an action *a* ∈ *A* that as well as possible trades off the (expected) utility of *a* relative to *r*′(*s*) = Resource‐Rational‐Belief(*C*,*s*,*A*) against the cost of applying *h*.

Anticipating that this new optimization problem is once again at least as hard as the previous, the authors propose that a meta‐heuristic for choosing heuristics operates as well (Lieder & Griffiths, 2019, p. 16; see also Lieder & Griffiths, 2017). This heuristic presumably shares the feasibility‐supporting features of the lower level heuristics, and it works so as to yield an approximately optimal overall trade‐off between accuracy and effort. This foreshadows the AT program's alternative proposal, to which we turn next.

While RR takes the unconstrained nature of classical optimization to be the fundamental problem, AT takes merely qualifying the optimization assumption to be a losing strategy for ensuring tractability. AT shuns all appeals to optimization, arguing that cognition is not optimized and does not necessarily perform optimally in any sense. Instead, organisms satisfice, performing just well enough (Simon, 1956).

Heuristics take center stage in AT; they are the starting point, rather than a late addition. The key claim is that organisms rely on an “adaptive toolbox” of simple heuristics (Gigerenzer, 2000; Gigerenzer et al., 2011). These heuristics are by their nature easy to employ. As with RR, it is recognized that determining which heuristic to apply in any given case is perhaps the more difficult problem (Wallin & Gärdenfors, 2000). Again, however, they can posit a simple meta‐heuristic for selecting the heuristics. The claim is modest: These heuristics generally lead to decent decisions. The new problem can again be broken into two steps:

Ecologically Rational Action

*Input:* A representation *r*(*s*) ∈ *R* of a situation *s* ∈ *S* and decision process *h* which is the output of Ecologically Rational Toolbox.

*Output*: An action *a* ∈ *A* which is “ecologically rational” because produced by *h* (i.e., often enough good enough).

Ecologically Rational Toolbox

*Input:* A representation *r*(*s*) ∈ *R* of a situation *s* ∈ *S* and a set *H* of building blocks which can be used to construct heuristics for producing actions.

*Output*: A toolbox of heuristics *T* constructed from elements of *H* which is “fast, frugal, and good enough” for situations like *s*.

AT is then designed to avoid CR and RR's problems. Organisms do very simple things that lead to success only because they are contextually appropriate. How “ecologically rational” agents are and how well any particular heuristic does in any particular environment are empirical questions (see, e.g., Goldstein & Gigerenzer, 2002). So AT's methodology is precisely the opposite of RR's, which uses strong rationality assumptions to derive empirical hypotheses; AT asks about rationality only once the descriptive facts have been laid out.

MM is closely related to AT, but it is premised on a different diagnosis of CR's intractability problem, which gives it a different character. It emphasizes CR's domain generality rather than its optimality (Fodor, 1983). A variety of more specific views fall under the heading of MM, and the essential characteristics of the “modules” responsible for cognition are still a matter of debate (cf. Barrett & Kurzban, 2006; Carruthers, 2003; Frankenhuis & Ploeger, 2007; Samuels, 2005, for discussion). We take the domain specificity and informational encapsulation of cognitive processes to be core commitments of MM (Samuels, 2012). Some proponents replace the requirement of informational encapsulation with the weaker requirement of functional specialization, but we set such views aside. For one, as Samuels explains, this move “renders MM more plausible… at the risk of leaching the hypothesis of its content, thereby rendering it rather less interesting than it may initially appear to be” (Samuels, 2012, p. 64). This is because it is not really controversial that the mind contains functionally specialized mechanisms. More importantly, we are interested in MM as a strategy for ensuring the tractability of cognition, and the guarantee of tractability is lost when information encapsulation is given up.^7^ Nonetheless, our formal interpretation of informational encapsulation (p. 11) is consistent with a variety of views (cf. Carruthers, 2003), and it seems to be a minimal requirement of modularity.

There is broad support for the view that the mind has some modules (such as for visual processing). As argued by Fodor, however, modularity is thought to be the only way to ensure tractability. As Carruthers puts it, tractability considerations

[dictate] that cognition should be realized in a system of encapsulated modules. You only have to begin thinking in engineering terms about how to realize cognitive processes in computational ones to see that the tasks will need to be broken down into separate problems which can be processed separately (and preferably in parallel). (2003, p. 505)

The mind therefore must contain distinct modules, each performing domain‐specific computations.^8^ In fact, some advocates of modularity (such as Fodor, famously [2001]) do not think that *all* of cognition can be modular. Since we are here concerned with attempts to explain how cognition can be tractable, however, we restrict our attention to the kind of “strong” MM which does insist that cognition is entirely modular. Notably, this includes so‐called higher or central processes, such as those involved in reasoning (Samuels, 2012).

As with AT, the divide‐and‐conquer cognitive architecture is meant to ensure that the mind's tasks are all relatively simple, and the architecture as a whole is tractable‐by‐design. In other words, MM poses the following problems:

Massively Modular Action

*Input:* A representation *r*(*s*) of situation *s* ∈ *S* and architecture *M* which is the output of Massively Modular Architecture.

*Output*: An action *a* ∈ *A* that is fitness promoting^9^ if *s* ∈ *S*′, where *S*′ is the set for which *M* was adapted.

Massively Modular Architecture

*Input:* A set of ecologically relevant situations *S*′ and a set of possible modular architectures M.

*Output*: A modular architecture M∈M which has good adaptive value for situations in *S*′.

Indeed, the *massive* part of the MM position is both the controversial part and the part for which the tractability argument is especially important (see, e.g., Okasha, 2003; Samuels, 2005). The task has therefore been seen as explaining how sophisticated cognition could be realized through the coordinated action of simple modules alone. (Observe the parallel with AT's challenge of explaining how heuristics could be matched to the right environments.) Although Fodor himself saw no real possibilities for solving this task (Fodor, 2001), solutions have been proposed (see e.g., Barrett, 2005; Carruthers, 2003).

### 2.2 The problems posed to evolution

Evolution looms large in all three of these research programs. It is responsible for producing the heuristics posited by RR and AT, and the modules posited by MM. In each case, evolution is credited with avoiding the intractability problem by yielding a tractable architecture. Of the problems formulated in Section 2.1 to characterize the three approaches, each approach poses problems both to cognition and to evolution. We next look at each approach in more detail.

Recall that RR posed new problems—Resource‐Rational Belief and Resource‐Rational Heuristic—in response to the critique of constrained optimization. Notice, however, that they shift the burden of solving these new problems to evolution:

[W]e propose that people never have to directly solve the constrained optimization problem… Rather, we believe that for most of our decisions the problem of finding a good decision mechanism has already been solved by evolution. (Lieder & Griffiths, 2019, p. 17)

Similarly, AT poses problems to both cognition and evolution: Ecologically Rational Action is to be solved by the mind, whereas Ecologically Rational Toolbox is to be solved by evolution. Evolution is responsible for configuring the AT, determining which heuristic should be used in which situation. This is what makes the toolbox *adaptive*.

MM has its roots in evolutionary psychology, so evolution is obviously central. MM proponents combine this with an engineering mindset, echoing RR. This is reflected in the fact that MM poses a straightforward set of problems: Evolution solves Massively Modular Architecture, and the mind uses that architecture to solve Massively Modular Action.

We can compare the roles of the three approaches' appeals to evolution using Cummins's (2000) distinction between strong and weak evolutionary programs in cognitive science. RR and AT include weak evolutionary programs, in that evolutionary considerations are meant to make more plausible cognitive architectures with independent motivation. Neither of these approaches engages in sophisticated theorizing about evolution. In contrast, MM has a strong evolutionary program. It is the direct product of evolutionary considerations: Modules are argued to be the kind of architecture that could evolve, even independent of tractability considerations (see, e.g., Cummins & Cummins, 1999, for discussion). For all three approaches, however, the appeal to evolution is important because evolution is taken to be the most plausible candidate for doing the extremely hard job of allowing agents to choose rationally, despite the intractability challenge.

## 3 Tractable‐by‐design is intractable to evolve

### 3.1 Generalized adaptation and its special cases

**Fig. 1:**
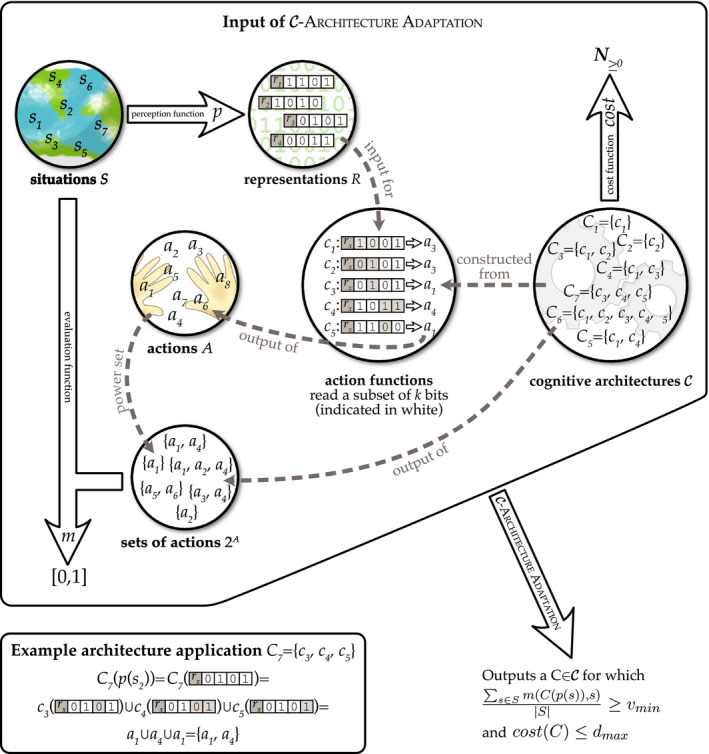
The C‐Architecture Adaptation problem. Circles represent sets, large arrows represent functions (or mappings), and dashed lines represent other relationships. The output of the adaptation process is in the bottom‐right corner. The box in the lower‐left corner illustrates how a particular (generic) cognitive architecture *C*_7_ can be applied to a representation of a situation *p*(*s*_2_).

RR, AT, and MM all aim to dissolve the apparent intractability of rational action at the cognitive level by assuming that the cognitive architecture is tractable‐by‐design. Intuitively, tractable cognitive architectures may seem to be “easier” or “more likely” to evolve than intractable ones. We present a set of proofs, however, that contradict this intuition. For constructing the proof argument it is useful to cast a generalized adaptation problem. We give a definition of such a problem below and present a visual illustration in Figure 1.

C‐Architecture Adaptation

*Input:* A set of relevant situations *S*; a set of possible representations *R*; a set of possible actions *A*; a perception function *p*: *S* → *R*, mapping situations to representations; an evaluation function *m*: 2*^A^* × *S* → [0,1], mapping combinations of actions and situation pairs to values; a class of architectures C (where each C∈C is a set of action‐selection functions *C* = {*c*_1_, *c*_2_, …, *c_l_*}, where each *c_i_*: *R* → *A* and *C*: *R* → 2*^A^* maps *l* ≥ 1 representations to *l* ≥ 1 actions, and has an associated *cost*(*C*) ∈ **N** ≥ 0); and two threshold values *v*_min_> 0 and *d*_max_ ≥ 0.

*Output*: A cognitive architecture C∈C such that $\frac{\sum_{s\in S}m\left(C\left(p\left(s\right)\right),s\right)}{\left|S\right|}\geq v_{\min}$ and *cost*(*C*) ≤ *d*_max_.

We briefly explain and motivate the different elements of this generalized adaptation problem and explain how the computational problems posed to evolution by RR, AT, and MM can each be seen as special cases^10^ of C‐Architecture Adaptation. Unless otherwise noted, the reader can assume that *cost*(*C*) = 0 for all *C* (variable costs are only used in RR).

One can think of *p*: *S* → *R* as a perception function, which could be many‐to‐one: Some situations may be perceptually indistinguishable for an organism. For simplicity and illustration purposes, we will assume that representations in *R* are binary strings. This is a high level of abstraction and without loss of generality, since any computable representation can be encoded as a binary string. Furthermore, we assume that *p* is fixed, as we are concerned only with the adaption of *cognitive* and not perceptual architecture. High‐level perception is arguably part of cognition (Chalmers, French, & Hofstadter, 1992), but for the purpose of our argument, we imagine that the agent has it for free. Note that additionally evolving the perception function cannot make evolution's problem easier.^11^

One can think of *m*: 2*^A^* × *S*→[0,1] as either an externalist evaluation function leading to real‐world outcomes with beneficial or detrimental consequences for the agent (e.g., for AT and MM) or as an internalist quality assessment function (e.g., for RR and CR). The values *m*(*a*_1_, … *a_l_*, *s*) denote how good actions *a*_1_,…*a_l_* are for a given situation *s*. Intuitively, *m*(*a*_1_, … *a_l_*, *s*) = 0 denotes that actions *a*_1_, … *a_l_* (combined) are a maximally poor choice for situation *s*, and *m*(*a*_1_,…*a_l_*, *s*) = 1 denotes that they are a maximally good choice. (It is possible to define the class C such that *l* = 1, i.e., that only one action is selected per situation.) The value *v*_min_ denotes the minimum *average* action quality that would count as “rational.” We require only that quality cannot be 0. Hence, the formalization applies both to notions of rationality that require optimality (i.e., *v*_min_ = 1 or is otherwise maximized, e.g., relative to *S*, *p*, *m* and C), and notions that merely require actions to be “good enough” on average (i.e., 0 < *v*_min_ << 1; cf. Rich et al., 2019).

**Fig. 2:**
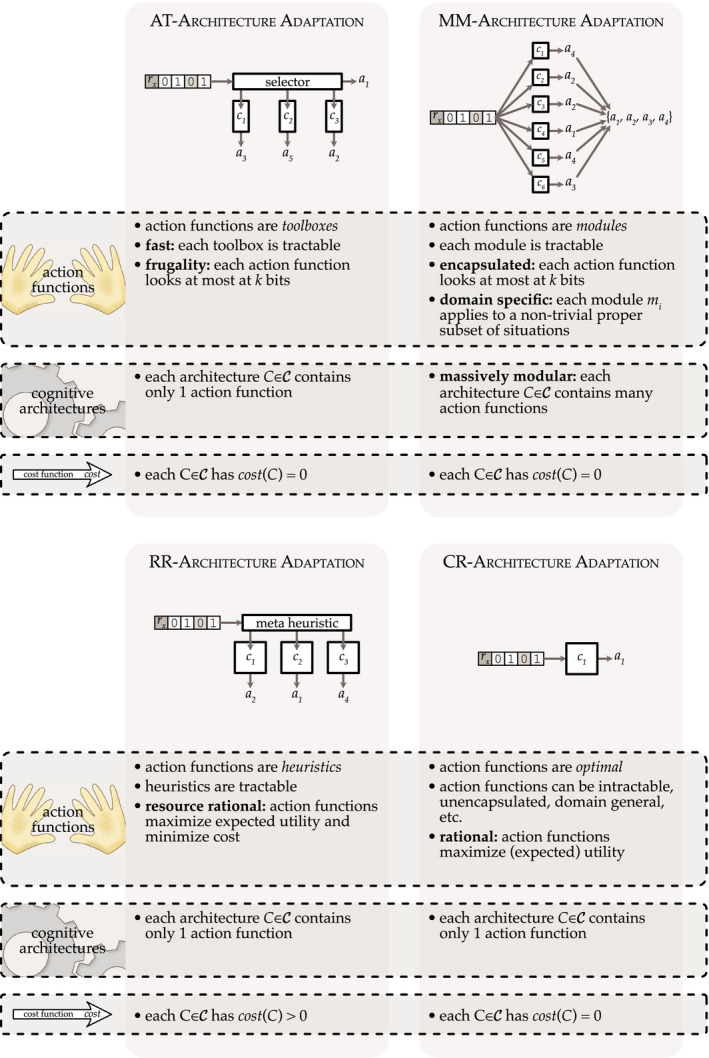
Four special cases of C‐Architecture Adaptation and their assumptions.

One can think of *S*, *p*, and *m* as the (experienced) environment to which the architecture C∈C is to be adapted. Here, *C* is modeled as a set of action‐selection functions *C* = {*c*_1_, *c*_2_, …, *c_l_*}, where each *c* ∈ *C* selects an action *a* ∈ *A* for a given perceived situation *p*(*s*) ∈ *R*.^12^ With these degrees of freedom we can define C such that it embodies the constraints specific to the different cognitive architectures postulated by AT, MM, and RR (see Figure 2). The explanatory strategy employed by the three approaches is to hold evolution responsible for configuring these action functions according to the constraints described below.

Regarding AT, we assume that *C* = {*c*} consists of a single action‐selection function that is tractably computable by a toolbox of fast and frugal heuristics. To ensure tractability of *C*, we assume that the heuristics are chosen by a meta‐heuristic which is itself fast and frugal (cf. Otworowska, Blokpoel, Sweers, Wareham, & van Rooij, 2018; Rich et al., 2019). Frugality, the assumption that a lot of information is ignored when making decisions, is modeled by the constraint that that function *c* ignores all but *k* << *n* bits of representations in *R*. Fastness is modeled by the constraint that *C*(*p*(*s*)) can be computed for each *s* ∈ *S* in a time that scales no faster than linearly (or otherwise as a low‐order polynomial) with the size of the toolbox.

Unlike AT, MM postulates a large number of modules (*massive* modularity), each activated by a proper subset of all possible situations (*domain specificity*), that may be activated in parallel and may even produce multiple actions in parallel. Hence, a MM cognitive architecture is a set *C* = {*c*_1_, *c*_2_, …, *c_l_*}, with *l* > 1. While each *c_i_* ∈ *C* is assumed to be *informationally encapsulated*—modeled by the constraint that each individual function *c_i_* ignores all but *k* << *n* bits of representations in *R*—the modules in combination may be able to access all bits in representations in *R*. This contrasts with AT's assumption that the entire cognitive architecture is frugal. Even though the number of simultaneously operational modules can be large, we assume it is upper bounded by $\frac{\left|S\right|}{b}$, with *b* ≥ 3. This constraint is to prevent architectures that are essentially huge “look‐up tables” where each module codes actions for a very small number of concrete situations. It is reasonable to assume that even though modules are domain specific and plentiful, each module will be activated in more than a handful of specific situations. Modules are instead activated in a large number of situations belonging to the subclass of situations defining their “domains.” This is in keeping with examples of modules such as “cheater detection,” which is activated in a relatively broad class of social situations, rather than a specific few (Cosmides, 1989).

RR architectures *C*, like AT architectures, contain a single action‐selection function, that is, *C* = {*c*}. Unlike AT, in RR architectures, the function *c* is tractably computable by a *resource‐rational* heuristic (possibly selected by a resource‐rational meta‐heuristic) relative to a *resource‐rational* belief about the state of the world. This means that both the action‐selection heuristic and the perception function trade off computational quality (of decision making and representation, respectively) against computational cost. Since in C‐Architecture Adaptation the perception function *p* is taken to be fixed, we simply assume that *p* is resource‐rational. This means that our computational complexity analysis of the problem (in Section 3.2) will give a lower bound on the true complexity, which would involve adapting *p* along with *C*. The distinct feature of RR is that the cost function *cost*(*C*) assigns different values for different C∈C.

So far we have shown that adapting AT, MM, and RR architectures can each be seen as a special case of C‐Architecture Adaptation, with different constraints on the class C. We close this subsection by observing that adapting classical architectures can also be seen as a special case of C‐Architecture Adaptation. Classically rational (CR) architectures assume that *C* = {*c*}, where *c* is a (expected) utility maximizing action‐selection function. The requirement of (expected) utility maximization can be modeled by setting *v*_min_ to the maximum value possible for the (experienced) environment *S*, *p*, and *m*. Figure 2 presents a visual illustration and overview of the different special cases of the C‐Architecture Adaptation problem.

### 3.2 Complexity theoretic results

**Fig. 3:**
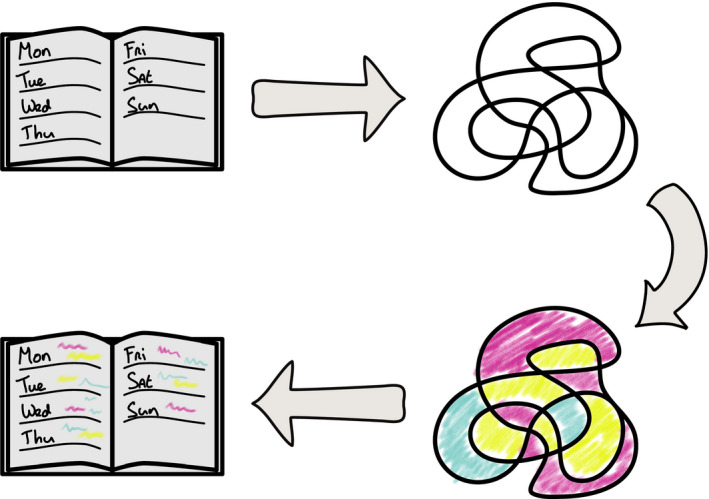
An intuitive illustration of the mathematical technique that we use for proving intractability. See main text for an explanation.

Before presenting our formal results, we provide a fictitious illustration to help make intuitive our formal proofs, their assumptions and implications. Consider a map coloring puzzle as in Figure 3. A child with three different crayons (say, pink, blue, and yellow) may try to color each area such that no two adjacent areas have the same colors. This looks like a fun and not prohibitively complex puzzle. In contrast, a parent regularly encounters a much less fun and more resource‐demanding problem, viz., the task of scheduling activities (meetings at work, shopping, cooking, dental appointments, etc.) while taking various constraints into account (deadlines, others' schedule constraints, school hours, etc.). Now imagine that the parent would discover an *easy* way to translate her difficult scheduling tasks into coloring puzzles such that a given scheduling task would correspond to a particular puzzle *and* a successful coloring of that map could be *easily* translated back into a constraint‐satisfying schedule. Then the parent could use the child's coloring activity as a way of solving her own hard scheduling tasks!

But now note the following: Existence of an easy translation from scheduling tasks to coloring puzzles means that if map coloring were easy, then scheduling would be, too. Namely, the parent would be able to solve all her scheduling problems tractably by translating scheduling tasks into coloring puzzles and then “reading off” the solutions from the child's colorings. Hence, if scheduling is intrinsically hard, then so is map coloring.

This example is less fictitious than it may seem. In fact, it is possible to easily translate scheduling tasks into map coloring puzzle by a mathematical proof technique called *polynomial‐time reduction* (Arora & Barak, 2009). Also, it is known that scheduling is an intractable problem (NP‐hard; Garey & Johnson, 1979, SS11 in Section A.5). Therefore, map coloring is also intractable (NP‐hard; Garey, Johnson, & Stockmeyer, 1974). In other words, map coloring is much harder than it may look, and as intrinsically hard as scheduling. No child, or anyone, has the capacity to tractably solve every possible coloring puzzle.

Using this proof technique, we show that adapting some classes of cognitive architectures are also intractable problems. Instead of scheduling, we use a known NP‐hard problem called Exact Cover by 3‐Sets (X3C; Garey & Johnson, 1979). Like the translation from scheduling to map coloring in our example, there exists tractable translations from X3C to each of:

C‐Architecture Adaptation with

- some C (Theorem 1).
- C = {*C*|*C is an adaptive toolbox*} (Theorem 2).
- C = {*C*|*C is a massively modular architecture*} (Theorem 3).
- C = {*C*|*C is a resource*‐*rational architecture*} (Theorem 4).

This establishes that adapting AT, MM, and RR architectures is intractable.

Next we show that, in contrast, adapting CR architectures is not intractable. Intuitively, this is because there is a straightforward computational strategy for selecting classically rational actions, namely to generate and then exhaustively search through all combinations of actions and currently possible situations, and then to select the action with maximum value. Since there is a fixed, constant‐sized description of the algorithm carrying out this process, it would be computationally tractable to provide an agent with some program for this algorithm. We therefore have the following complexity theoretic result (for the formal proof and more details, see the Supplementary Materials):

C‐Architecture Adaptation is tractable for:

- C = {*C*|*C is an classically rational architecture*} (Theorem 5).

In sum, we have the—possibly counterintuitive—conclusion that adapting various tractable architectures is intractable, while adapting an intractable architecture is tractable.

## 4 Conclusion

We discuss the conceptual interpretation of the (in)tractability results presented in Section 3. These results establish that there exist *no* algorithms that can reliably solve the adaptation problems posed by RR, AT, and MM, using a reasonable amount of (polynomial) resources.^13^ In contrast, CR is tractable to evolve. This does not rule out that CR's evolution would have been impossible for other reasons, but it does show that the tractable‐by‐design approaches have not shown themselves to be superior with respect to the tractability challenge.

The state of affairs is intuitively this: The original Rational Action problem is simply extremely hard, and it is not clear how agents solve it. There is no easy way to make this hardness go away. RR, AT, and MM make the problem easy at the cognitive level only by pushing the hard work to the evolutionary level—but the work remains hard. In contrast, CR holds cognition responsible for the hard work. This leaves evolution with relatively little to do; it must simply select general‐purpose problem‐solving abilities.

One might object that evolution is not deterministic, perfect, or goal‐oriented. Importantly, we do not assume that it is. We take biological evolution to be a randomized process of mutation, reproduction, and selection, as widely accepted among evolutionary biologists. Since the generative and search abilities of non‐deterministic, randomized search processes cannot exceed the limits of deterministic tractable computation,^14^ computational complexity constraints apply to evolution just the same (see also Kaznatcheev, 2019; Valiant, 2009). This constraint on randomized computation is general. Hence, allowing biological evolution to interlace with other randomized and/or teleological processes, such as cultural evolution (Boyd & Richerson, 1988; Mesoudi, 2016), does nothing to remove this limitation.

Similarly, one might imagine that adjusting particular features of the formalism would result in a different verdict regarding tractability. In our experience, such intuitions are easily disproven through minor adjustments to the formalization and proofs; see, for example, Otworowska et al. (2018) and Rich et al. (2019), which provide related results pertaining to AT. The latter paper in particular addresses common misunderstandings of results of this kind in great detail.

We close by noting that although our results are mainly negative, they are not meant to discourage research into any particular approach to cognition, but rather to encourage work toward providing genuine—and not merely apparent—solutions to the intractability problem. In terms of what such solutions might look like, we can only speculate. If we were required to bet, however, we would bet against the idea that evolution has made all of the cognition tractable‐by‐design, because this shifts too much responsibility to evolution. Instead, evolution more plausibly would have done just part of the work, preparing cognition to flexibly configure its own action‐selection strategies. How could architectures that are not tractable‐by‐design nonetheless support action selection that is both rational and tractable for our world? Much previous research suggests that the world must be somehow “friendly”—and perhaps friendly to both cognition and to evolution—in that it tends to present tasks that can be solved (Rich et al., 2019; van Rooij, Blokpoel, de Haan, et al., 2019; van Rooij et al., 2018). This is still not an explanation, but shows directions in which explanations might be sought. In the end, our intuition cannot take us very far; only formal tractability analyses relative to different assumptions can establish whether a given hypothesis overcomes the tractability challenge.

## Supporting information

**Supplementary Material**.

## Notes

^1^ For ease of exposition, we do not explicitly represent the utility function for actions.

^2^ There are reasons to doubt the viability of attempts to salvage the approach; see, e.g. Kwisthout, Wareham, & van Rooij, 2011; van Rooij, Wright, Kwisthout, & Wareham, 2018; van Rooij, Wright, & Wareham, 2012.

^3^ While Gigerenzer (this issue) criticizes computational‐level analyses, ours are not of the type he argues against; as will become clear, his preferred explanation of cognition can also be stated at the computational level.

^4^ On an alternative interpretation, the computational level may go beyond the causal level. There is room for disagreement, since our argument does not depend on our preferred characterization of the levels.

^5^ We thank Niels Taatgen for pointing this out.

^6^ That is, a belief: “Each computation *c* ∈ *C* costs time and energy… but may improve the system’s decision by changing its belief *B*” (Griffiths et al., 2015, p. 224).

^7^ In describing an "enzymatic" account of MM, for example, Barrett notes that “enzymes are Turing Universal” (2005, p. 268). But if mental modules function analogously to enzymes with such computational power, then the cognitive system is no longer tractable‐by‐design.

^8^ An example of a module is “cheater detection”: In Wason's well‐known selection task, subjects typically fail to correctly apply modus tollens to abstract content, but perform well when the task can be construed as checking whether a norm is violated. This performance difference is taken as evidence of domain‐specific cognitive processes (Cosmides, 1989; Gigerenzer & Hug, 1992; but see Oaksford & Chater, 1994; Stenning & van Lambalgen, 2001).

^9^ Intuitively, “fitness” measures the reproductive prospects of the agent. The behavior should count as successful given the environment. The notion of fitness is actually problematic, since the reproductive value of a trait is often highly contingent, not stable (see e.g., Okasha, 2018, Ch. 3). This complication does not affect our argument, though it points toward yet another serious problem with many appeals to evolution.

^10^ Formally, a computational problem (input–output mapping) *F_S_*:*I_S_* → *O_S_* is a *special case* of a computational problem *F*:*I* → *O*, iff *F*(*i*) = *F_S_*(*i*) for all *i* ∈ *I_S_*, and *I_S_* ⊂ *I*.

^11^ This is true unless the intractable cognitive work is smuggled into the perception function (cf. Theorem 5 in Section 3.2), which would be cheating.

^12^ Each *c_i_* is active in some proper subset of situations; these subsets may overlap. Although an active *c_i_* always chooses an action, non‐action can itself be an action.

^13^ The existence of such algorithms would contradict the famous conjecture in theoretical computer science that P ≠ NP (Fortnow, 2009); to our knowledge, this is not contested in the relevant debates.

^14^ Assuming BPP = P and/or RP ≠ NP (Arora & Barak, 2009).
