# Supplementary material for: How Intractability Spans the Cognitive and Evolutionary Levels of Explanation
Source: Top Cogn Sci. 2020 Jun 4;12(4):1382–402. doi: 10.1111/tops.12506 (PMC7687229; doi:10.1111/tops.12506)
Supplement: Supplementary file 1 [file TOPS-12-1382-s001.pdf]

Supplementary materials for ‘How Intractability Spans the Cognitive and Evolutionary Levels of Explanation’

Patricia Rich[1,2], Mark Blokpoel[3], Ronald de Haan[4], and Iris van Rooij[3]
[1] University of Hamburg, Philosophy Department
[2] University of Bayreuth, Philosophy Department
[3] Radboud University, Donders Institute for Brain, Cognition, and Behaviour
[4] Institute for Logic, Language and Computation, University of Amsterdam

1 Computational Complexity Basics

The proofs presented in these Supplementary Materials build on concepts and techniques from computational complexity theory. We briefly introduce the key ideas. For more detailed treatments we refer the reader to textbooks on the topic (e.g. Arora and Barak, 2009; van Rooij, Blokpoel, Kwisthout, and Wareham, 2019). It is implicit in the debates between the approaches we consider that computations that take more than some polynomial amount of resources (time, space, randomness) are intractable (Chater, Tenenbaum, & Yuille, 2006; Gigerenzer, Hoffrage, & Goldstein, 2008). We refer the reader to extensive discussion, including defenses and qualifications, of this view elsewhere (van Rooij et al., 2019; van Rooij, 2008).

Computational complexity theory distinguishes, among other things, between the classes P and NP. These are both classes of *decision* problems, i.e., problem with only *Yes* or *No* answers. Here is an example of a decision problem that we will be using in our proofs:

> Exact Cover by 3-Sets
> *Input:* A set U of $3n$ elements, and a family $\mathcal{F} = \{F_1, \dots, F_m\}$ of subsets of U, such that for each $1 \leq j \leq m$ it holds that $|F_j| = 3$.
> *Question*: Does there exist $\mathcal{F}' \subseteq \mathcal{F}$ such that $|\mathcal{F}'| = n$ and $\bigcup \mathcal{F}' = U$?

The class P is the class of decision problems that can be *solved* using a polynomial-time algorithm, i.e., an algorithm that takes on the order of n^c basic computational steps, for some constant c (also denoted as $O(n^2)$). The class NP is the class of decision problems with the property that if the answer is *Yes*, then there exist a proof that the answer is *Yes* (called a ‘witness’ or ‘certificate’) that can be *verified* in polynomial-time. It is known that $\mathsf{P} \subseteq \mathsf{NP}$, and conjectured that $\mathsf{P} \neq \mathsf{NP}$ (Fortnow, 2009). Since this conjecture is widely believed by computer scientists and mathematicians, and to the best of our knowledge none of our intended interlocutors question this conjecture, we assume it here and throughout the main text of the article.

Using the $\mathsf{P} \neq \mathsf{NP}$ conjecture it can be proven that some problems in (or outside) NP are not in P, and hence not polynomial-time solvable. Among them are the NP-hard problems. NP-hard problems have the property that if any one of them were to be polynomial-time solvable then $\mathsf{P} =$

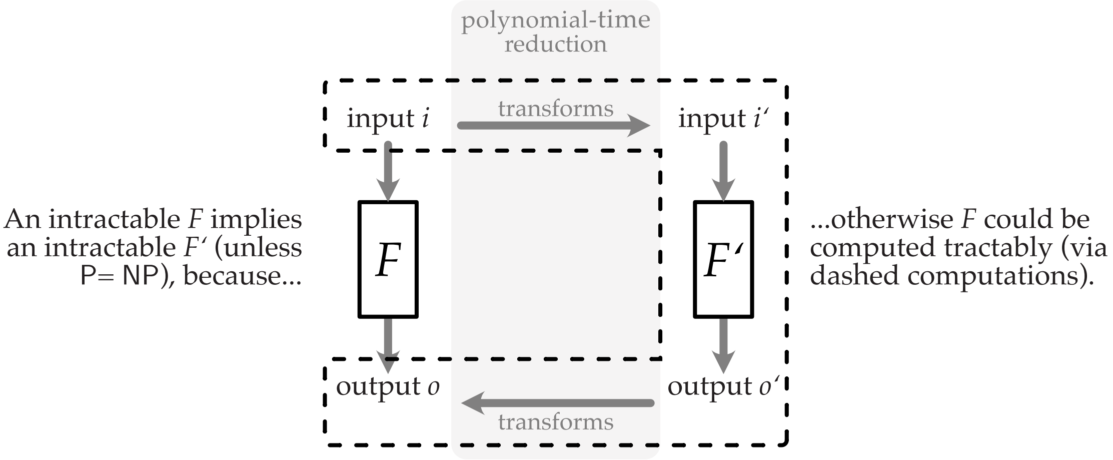

Figure 1: Proving (by contradiction) the intractability of F' via polynomial-time reduction from F.

NP (which would contradict the $\mathsf{P} \neq \mathsf{NP}$ conjecture). Hence, assuming $\mathsf{P} \neq \mathsf{NP}$ a problem can be shown to be intractable (i.e., not polynomial-time solvable) by proving it NP-hard.

A problem can be proven NP-hard using the technique of polynomial-time reduction. It works as follows: Let $F : I \to O$ be a known intractable (NP-hard) problem, and let $F' : I' \to O'$ be a new problem of interest. Then we say $'I$ *polynomial-time reduces* to F' if there exist two tractable (i.e., polynomial-time) algorithms $\mathbb{A}$ and $\mathbb{B}$ where $\mathbb{A}$ can transform any input $i \in I$ into an input $A(i) = i' \in I'$ such that $\mathbb{B}(F'(\mathbb{A}(i))) = F(i)$. Observe that if F' were to be tractable, then algorithms $\mathbb{A}$ and $\mathbb{B}$ could be used to tractably solve F. Since F is known to be intractable, a polynomial-time reduction from F' to F proves that F must be intractable too (see Figure 1 for an illustration).

Problems that are both NP-hard and in NP are called NP-complete. The example decision problem that we presented, Exact Cover by 3-Sets, is a known NP-complete problem (Garey & Johnson, 1979). We will use this decision problem as a starting point for our proofs. In a sense, it does not matter which problem we use, since it is known that every NP-complete problem can be polynomial-time reduced to every other NP-complete problem.

In the main text of the paper, we consider search problems rather than decision problems. That is, the problems are not concerned with answering a binary Yes-No question for a given input, but are concerned with computing a different output. For example, $\mathcal{C}$-Architecture Adaptation deals with the problem of computing an architecture $C \in \mathcal{C}$ with certain properties—if it exists.

Notions of intractability that are typically used in computational complexity theory are based on decision problems—e.g., NP-hardness. In the proofs that follow in this supplementary material (in Section 2), we use these hardness notions to show that search problems are not tractably solvable. We do this by showing that if (1) we could solve the search problems tractably, then (2) we could also solve some NP-hard decision problem in polynomial time. Formally, we show this implication by using what is called a polynomial-time Turing reduction.[1] This gives us as result the statement that we cannot solve these search problems tractably, unless $\mathsf{P} = \mathsf{NP}$.

[1] For a formal definition of Turing reductions—see, e.g., (Arora & Barak, 2009).

2 Proofs

Theorem 1. $\mathcal{C}$-Architecture Adaptation *is* NP*-hard.*

Proof. We show NP-hardness by a polynomial-time reduction from X3C, that works as follows. We take an input of X3C, and we use this input to construct an input of $\mathcal{C}$-Architecture Adaptation. Then, we show that we can use any polynomial-time algorithm for $\mathcal{C}$-Architecture Adaptation to decide in polynomial time whether the answer for the original input of X3C is "yes" or "no."[2]

Let $(U, \mathcal{F})$ be an instance of X3C, where $U = \{u_1, \dots, u_n\}$, where $\mathcal{F} = \{F_1, \dots, F_m\}$, and where $|F_j| = 3$ and $F_j \subseteq U$ for each $1 \leq j \leq m$. We construct an input of $\mathcal{C}$-Architecture Adaptation as follows.

We take the set $\{s_0, s_1, \dots, s_n\}$ of relevant situations, and we take the set $A = \{a_1, a_2\}$ of actions. All representations in R are binary strings of length m. The perception function $p : S \to R$ is defined as follows. We let $p(s_0) = 000\dots0$, i.e., the string consisting of m zeroes. For each $1 \leq i \leq n$, we let $p(s_i)$ be the string $r_{i,1} \dots r_{i,m}$, where $r_{i,\ell} = 1$ if $s_i \in F_\ell$ and $r_{i,\ell} = 0$ if $s_i \notin F_\ell$. We then define $m : 2^A \times S \to [0,1]$ as follows. We let $m(B, s_0) = 1$ if $B = \{a_2\}$ and $m(B, s_0) = 0$ if $B \in 2^A \setminus \{\{a_2\}\}$. Similarly, for each $1 \leq i \leq n$ we let $m(B, s_i) = 1$ if $B = \{a_1\}$ and $m(B, s_i) = 0$ if $B \in 2^A \setminus \{\{a_1\}\}$. In other words, for situation s_0, only the singleton set $\{a_2\}$ gives value 1, and all other sets of actions give value 0, and for all other situations s_i, only the singleton set $\{a_1\}$ gives value 1, and all other sets of actions give value 0. This construction is illustrated for an example instance of X3C in Figure 2.

$s \in S$	$p(s) \in R$	unique B s.t. $m(B, s) = 1$
s_0	00000	$\{a_2\}$
s_1	11000	$\{a_1\}$
s_2	10110	$\{a_1\}$
s_3	01101	$\{a_1\}$
s_4	10000	$\{a_1\}$
s_5	01011	$\{a_1\}$
s_6	00111	$\{a_1\}$

Figure 2: Construction of S, A, R, p and m in the proof of Theorem 1, for the example input $(U, \mathcal{F})$, where $U = \{u_1, \dots, u_6\}$, $\mathcal{F} = \{F_1, \dots, F_5\}$, $F_1 = \{u_1, u_2, u_4\}$, $F_2 = \{u_1, u_3, u_5\}$, $F_3 = \{u_2, u_3, u_6\}$, $F_4 = \{u_2, u_5, u_6\}$, and $F_5 = \{u_3, u_5, u_6\}$. In this example, the architecture $C_{\{1,5\}}$ achieves a maximal cumulative value for m.

As the class $\mathcal{C}$ of architectures, we consider the set of all singleton sets $C_L = \{c_L\}$ where $L \subseteq \{1, \dots, m\}$ is a set of size exactly $n/3$. Each such $c_L : R \to A$ is the function that is defined as follows. For $L = \{i_1, \dots, i_{n/3}\}$, the function c_L takes as input a binary string $r \in R$ of length m, and it returns a_1 if there is some $i_j \in L$ such that the i_j-th bit of r equals 1, and it returns a_2 otherwise. Moreover, each such $C_L = \{c_L\}$ has a fixed constant cost, say, $cost(C_L) = 0$. Finally, we let $v_{min} = 1$ and $d_{max} = 0$.

[2]Technically, this is what is called a *Turing reduction*—see, e.g., (Arora & Barak, 2009).

We now show that we can use any polynomial-time algorithm $\mathbb{A}$ for $\mathcal{C}$-Architecture Adaptation to decide whether there exists a subset $\mathcal{F}' \subseteq \mathcal{F}$ of size $|\mathcal{F}'| = n/3$ such that $\bigcup \mathcal{F}' = U$. We do so by running the algorithm $\mathbb{A}$ on the instance of $\mathcal{C}$-Architecture Adaptation that we constructed. This algorithm then either (i) outputs some $C_L \in \mathcal{C}$, or (ii) it outputs something else, e.g., the string "none found." We consider these two cases separately.

In case (i), we check whether $\sum_{s \in S} m(C_L(p(s)), s)/|S| \geq v_{min} = 1$. If this is the case (i.a), we know that in each situation $s \in S$, the architecture C_L outputs a set B of actions such that $m(B, s) = 1$. Then, by construction of the input, this can only be the case if $\mathcal{F}' = \{F_j \mid j \in L\}$ has the property that $\bigcup \mathcal{F}' = U$. Moreover, we also know that $|\mathcal{F}'| = n/3$. Thus, we can conclude that the answer to the original input for X3C is "yes."

Next, we show that in case (i.b), where $\sum_{s \in S} m(C_L(p(s)), s)/|S| < 1$, and in case (ii), where the algorithm $\mathbb{A}$ outputs something that is not in $\mathcal{C}$, the answer to the original input for X3C is "no." In both cases, the algorithm does not output an architecture C with the property that $\sum_{s \in S} m(C(p(s)), s)/|S| \geq v_{min} = 1$. We show that if the answer to the original input for X3C would be "yes," then there exists an architecture C with the property that $\sum_{s \in S} m(C(p(s)), s)/|S| \geq v_{min} = 1$, which contradicts our assumption that the algorithm $\mathbb{A}$ works correctly to solve $\mathcal{C}$-Architecture Adaptation.

Suppose that there is some $\mathcal{F}' \subseteq \mathcal{F}$ such that $|\mathcal{F}'| = n/3$ and $\bigcup \mathcal{F}' = U$. Then take the architecture C_L, where L contains all $1 \leq j \leq m$ such that $F_j \in \mathcal{F}'$. Then $|L| = n/3$, since $|\mathcal{F}'| = n/3$. Moreover, one can straightforwardly verify that in each $s \in S$ the architecture C_L outputs a set B of actions such that $m(B, s) = 1$. In other words, $\sum_{s \in S} m(C_L(p(s)), s)/|S| = 1$. This concludes our proof. ☐

Theorem 2. *$\mathcal{C}$-Architecture Adaptation is* NP*-hard for* $\mathcal{C} = \{C \mid C$ *is an adaptive toolbox*$\}$.

Proof. To prove this, we can directly use the proof of Theorem 1. The class $\mathcal{C}$ of architectures used in this proof is a subset of all architectures implemented by a toolbox that looks at at most $n/3$ cues. Moreover, all arguments in the proof carry through if instead we consider the class of all fast-and-frugal trees of size at most $n/3$ (each of which has constant cost). Therefore, this proof also shows that $\mathcal{C}$-Architecture Adaptation is NP-hard for $\mathcal{C} = \{C \mid C$ is an adaptive toolbox$\}$.

Note that the number $k = n/3$ grows linearly in the size of the number $|S|$ of relevant situations. However, by making copies of situation s_0 in the proof of Theorem 1, we can make the number k (i.e., the number of bits any given heuristic can access) much smaller than the number $|S|$ of relevant situations. ☐

Theorem 3. *$\mathcal{C}$-Architecture Adaptation is* NP*-hard for* $\mathcal{C} = \{C \mid C$ *is a massively modular architecture*$\}$.

Proof. To prove this, we use a modified version of the proof of Theorem 1. What we change in the proof of Theorem 1 is the following. We modify m, by letting $m(B, s_0) = 1$ if $B = \{a_2\}$ and $m(B, s_0) = 0$ if $B \in 2^A \setminus \{\{a_2\}\}$, and for each $1 \leq i \leq n$, letting $m(B, s_i) = 1$ if $B = \{a_1, a_2\}$ and $m(B, s_i) = 0$ if $B \in 2^A \setminus \{\{a_1, a_2\}\}$. In other words, for situation s_0, only the set $\{a_2\}$ gives value 1, and all other sets of actions give value 0, and for all other situations s_i, only the singleton set $\{a_1, a_2\}$ gives value 1, and all other sets of actions give value 0. Moreover, we introduce two copies s_0', s_0'' of situation s_0, that behave exactly like situation s_0.

Now, consider the set $C_{all} = \{c_0, c_1, \ldots, c_m\}$ of functions $c_i : R \to A$, where c_0 is the function that looks at the first bit, and always outputs $\{a_2\}$ (regardless of the value of the first bit), and where for each $1 \leq j \leq m$, the function c_j looks at the j-th bit, outputs the action a_1 if the bit

equals 1, and outputs the action a_2 if the bit equals 0. Then, as the class of architectures, we take $\mathcal{C} = \{C \mid C \subseteq C_{all}, |C| \leq |S|/3\}$. Each such $C \in \mathcal{C}$ has a fixed constant cost, say, $cost(C) = 0$.

All arguments in the proof of Theorem 1 carry through (in an analogous form) if we consider this modified construction and the adapted class $\mathcal{C}$ of architectures. Moreover, these architectures fit the conditions of massively modular architectures. Therefore, this proof also shows that Massively Modular Architecture Adaptation is NP-hard. □

Theorem 4. *$\mathcal{C}$-Architecture Adaptation is NP-hard for $\mathcal{C} = \{C \mid C$ is a resource rational architecture$\}$.*

Proof. To prove this, we use a modified version of the proof of Theorem 1. What we change from the proof of Theorem 1 is the following. As class $\mathcal{C}$ of architectures, we consider the set of all singleton sets $C = \{c\}$ such that c is any polynomial-time computable function $c : R \to A$. For each such $C = \{c\}$, the cost $cost(C)$ is the maximum number of bits of $p(s) \in R$ that c looks at in any situation $s \in S$.

By using arguments that are entirely analogous to the arguments used in the proof of Theorem 1 for this class $\mathcal{C}$ of architectures, we get that the answer to the original input of X3C is "yes" if and only if there is an architecture C of cost $n/3$ that has the property that $\sum_{s \in S} m(C(p(s)), s)/|S| \geq v_{min} = 1$. Moreover, by construction there is no architecture C of cost $< n/3$ that has the property that $\sum_{s \in S} m(C(p(s)), s)/|S| \geq v_{min} = 1$. Therefore, analogously to the arguments in the proof of Theorem 1, we can use any polynomial-time algorithm $\mathbb{A}$ for $\mathcal{C}$-Architecture Adaptation to decide X3C in polynomial time.

Since this class $\mathcal{C}$ of architectures fits the conditions of resource-rational architectures, this proof shows that if $\mathcal{C} = \{C \mid C$ is a resource rational architecture$\}$ then $\mathcal{C}$-Architecture Adaptation is NP-hard. □

Theorem 5. *$\mathcal{C}$-Architecture Adaptation is polynomial-time solvable for $\mathcal{C} = \{C \mid C$ is a classically rational architecture$\}$.*

Proof. We show that $\mathcal{C}$-Architecture Adaptation is polynomial-time solvable for $\mathcal{C} = \{C \mid C$ is a classically rational architecture$\}$ by describing a polynomial-time computable algorithm $\mathbb{A}$ that solves this problem. The algorithm $\mathbb{A}$ takes as input a description of the sets S, R and A. It also takes as input a description of the functions $p : S \to R$ and $m : 2^A \times S \to [0, 1]$—without loss of generality, we suppose that these functions p and m are given in the form of (a description of) Turing machines that compute these functions.

The algorithm $\mathbb{A}$ outputs an architecture $C = \{c\}$ that achieves a maximal value of $\sum_{s \in S} m(C(p(s)), s)/|S|$. The function $c : R \to A$ is computed by an algorithm $\mathbb{B}$—that does not necessarily run in polynomial time. This algorithm $\mathbb{B}$ does the following:

1. Take as input any $r \in R$.
2. Compute the subset of situations $S' \subseteq S$ that are represented as r, i.e., $\forall_{s \in S'} p(s) = r$. This is equivalent to computing $p^{-1}(r)$, where p^{-1} denotes the inverse of p.
3. Iterate over all actions in A selecting the action $a \in A$ that maximizes the value of $\sum_{s \in p^{-1}(r)} m(\{a\}, s)$.
4. Output a.

By doing so, $\mathbb{B}$ maximizes the value of $\sum_{s \in S} m(C(p(s)), s)/|S|$.

Given a description of the sets S, R and A, and a description of the algorithms that compute the functions m and p, algorithm $\mathbb{A}$ can in polynomial time construct a description of a Turing machine that computes algorithm $\mathbb{B}$ as given above. This is the case, because the algorithm consisting of steps (1)–(4) is always the same, modulo some input parameters—only S, R, A and the descriptions of algorithms computing m and p change. These changing parameters can easily be plugged into a pre-defined algorithm in polynomial time. By doing so, $\mathbb{A}$ solves the problem $\mathcal{C}$-ARCHITECTURE ADAPTATION in polynomial time for $\mathcal{C} = \{C \mid C \text{ is a classically rational architecture}\}$. □

References

Arora, S. & Barak, B. (2009). *Computational complexity – a modern approach.* Cambridge University Press.

Chater, N., Tenenbaum, J. B., & Yuille, A. (2006). Probabilistic models of cognition: Conceptual foundations. Elsevier.

Fortnow, L. (2009). The Status of the P Versus NP Problem. *Communications of the ACM*, *52*(9), 78–86.

Garey, M. R. & Johnson, D. R. (1979). *Computers and intractability.* San Francisco: W. H. Freeman and Company, New York.

Gigerenzer, G., Hoffrage, U., & Goldstein, D. G. (2008). Fast and frugal heuristics are plausible models of cognition: Reply to Dougherty, Franco-Watkins, and Thomas (2008). *Psychological Review*, *115*(1), 230–239.

van Rooij, I., Blokpoel, M., Kwisthout, J., & Wareham, T. (2019). *Cognition and intractability: A guide to classical and parameterized complexity analysis.* Cambridge: Cambridge University Press.

van Rooij, I. (2008). The tractable cognition thesis. *Cognitive science*, *32*(6), 939–984.
