# Supplementary material for: How Intractability Spans the Cognitive and Evolutionary Levels of Explanation
Source: Top Cogn Sci. 2020 Jun 4;12(4):1382–402. doi: 10.1111/tops.12506 (PMC7687229; doi:10.1111/tops.12506)
Supplement: Supplementary file 2 — Supplementary Material. [file TOPS-12-1382-s002.pdf]

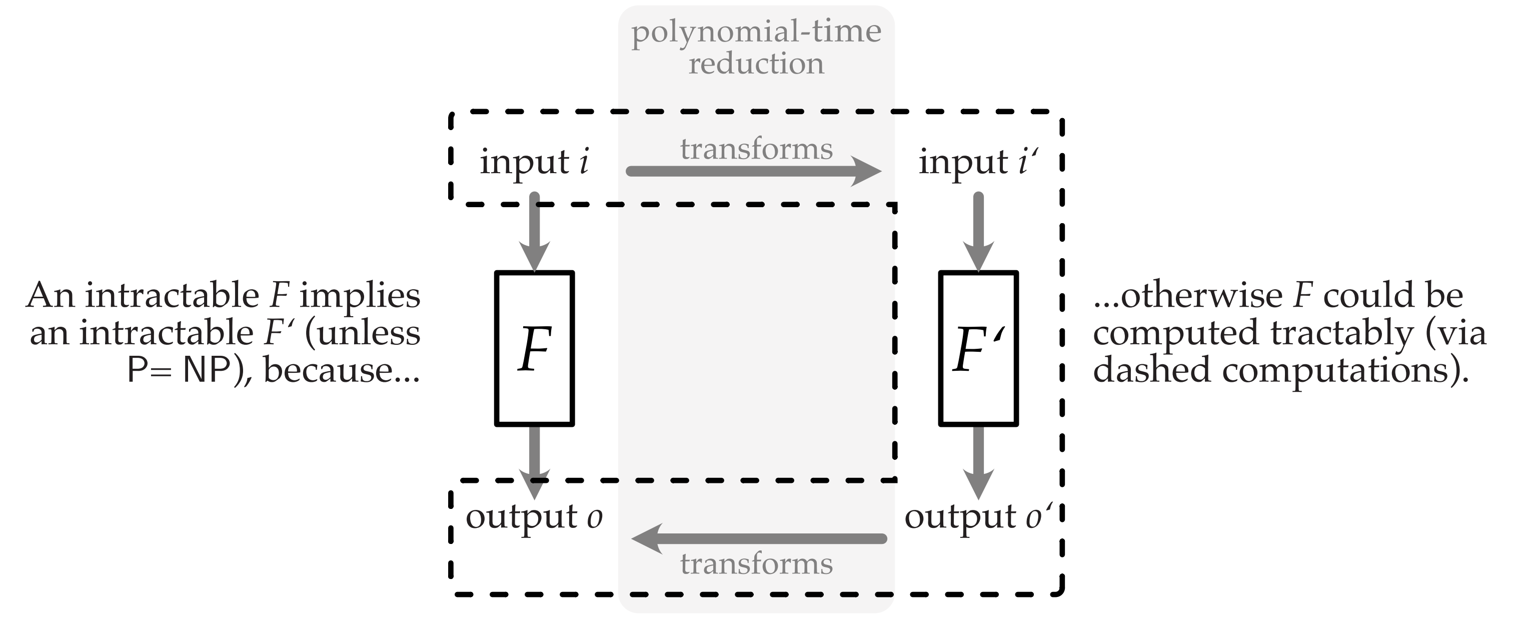
polynomial-time reduction
input i
transforms
input i'
An intractable F implies an intractable F' (unless P= NP), because...
F
F'
...otherwise F could be computed tractably (via dashed computations).
output o
output o'
transforms
